# *De novo* abnormalities identified by fluorescence *in situ* hybridization during follow-up confer poor prognosis in Chinese multiple myeloma

**DOI:** 10.3389/fmed.2025.1536825

**Published:** 2025-03-05

**Authors:** Shumin Chen, Lu Gao, Lin Feng, Zheng Wang, Ye Li, Qing Liu, Wenjie Song, Shu Kong, Yang Liu, Jin Lu, Yingjun Chang, Xiaojun Huang, Yueyun Lai

**Affiliations:** Beijing Key Laboratory of Hematopoietic Stem Cell Transplantation for Hematological Diseases, National Clinical Research Center for Hematologic Disease, Peking University Institute of Hematology, Peking University People's Hospital, Beijing, China

**Keywords:** multiple myeloma, survival, high-risk, clonal evolution, cytogenetics

## Abstract

**Background:**

Although there is evolving consensus to re-evaluate cytogenetic features during follow-up in multiple myeloma (MM), longitudinal studies on cytogenetic evolution in Chinese MM patients are still lacking. Our aim was to highlight the importance of ongoing monitoring of cytogenetic characteristics and shed light on the implications of clonal evolution in Chinese MM patients.

**Patients and methods:**

The clinical data of 230 MM patients were retrospectively analyzed, including 100 patients were continuously monitored for cytogenetic abnormalities by fluorescence *in situ* hybridization (FISH).

**Results:**

49 out of 100 patients acquired *de novo* FISH abnormalities during follow-up, which were associated with disease progression (*p* = 0.003) and inferior progression free survival (PFS) (median 31 vs. 51 months, *p* = 0.032). Patients with ≥2 *de novo* FISH abnormalities had poorer PFS (median 24 vs. 45 months, *p* = 0.003) when compared to those with l or no *de novo* FISH abnormality. Patients who acquired new abnormalities within 31 months since diagnosis had significantly worse PFS (median: 20 vs. 41 months, *p* < 0.001) and Overall Survival (OS) (median: 61 vs. 100 months, *p* = 0.008) compared to those who acquired new abnormalities after 31 months. When gain/amp *1q21*, *del(17p)*, t(4;14), and t(14;16) were classified as high risk abnormalities (HRA), patients with ≥2 HRA had a shorter PFS (median 28 vs. 49 months, *p* = 0.038) and OS (median 75 vs. 107 months, *p* = 0.040) when compared to those without HRA.

**Conclusion:**

Re-evaluation of cytogenetic characteristics by serial FISH tests is important in MM patients. *De novo* FISH abnormalities during follow-up are adverse prognostic factors, especially when ≥2 new FISH anomalies and acquired new abnormalities within 31 months since diagnosis are presented, and the presence of ≥2 HRA during the disease process are associated with poor survival in Chinese MM patients.

## Introduction

Multiple myeloma (MM) is the second most commonly diagnosed hematological malignancy, characterized by the proliferation of malignant plasma cells in the bone marrow and excessive production of immunoglobulins (1, 2). In recent years, as new therapies including immunomodulators (IMiDs), proteasome inhibitors (PIs) and monoclonal antibodies (mAbs) have been incorporated into standard treatments, the overall survival (OS) and progression-free survival (PFS) of MM have been significantly improved. However, most cases still remain a chronic and incurable disease due to its typical pattern of remission and relapse (3–6). Heterogeneous cytogenetic abnormalities are the most important characteristics of MM and cytogenetic analysis is essential for prognostic evaluation at diagnosis (7). Many studies had identified that some cytogenetic abnormalities including *del(17p)(p53)*, t(4;14)(p16;q32), t(14;16)(q32;q23), and t(14;20)(q32;q12) were high-risk abnormalities (HRA) in MM patients, and others such as *del(13q)* and t(11;14)(q13;q32) were considered as standard-risk factors, whereas the prognostic value of *1q21* gain/amplication (gain/amp *1q21*) had been controversial (8–13). Of note, most of the previous studies mainly focused on the prognostic impact of the abnormalities identified at diagnosis, only few studies had considered the significance of the new acquired cytogenetic aberrations throughout the course of the disease (14–16). A longitudinal cytogenetic study focusing on cytogenetic evolution of 128 patients from the time of primary diagnosis and at relapse from Merz et al. (17) revealed that the presence of a new acquired HRA during follow-up conferred to poor prognosis as well. The study from Binder et al. (7) showed that the development of additional abnormalities during the 3 years following diagnosis was associated with increased subsequent mortality. While these previous studies had highlighted the importance of ongoing monitoring of MM cytogenetic signatures, they were not sufficient to adequately assess all potentially HRA that occur during the disease process in the case of modern therapies. For example, it is unclear whether HRA emerged at diagnosis or during follow-up has different effects on the outcome of MM patients. In addition, longitudinal studies on cytogenetic evolution in Chinese MM patients are still lacking. Therefore, in the present study, we summarized the clinical data of 230 newly-diagnosed MM (NDMM) patients admitted to our hospital, focusing on the analysis of 100 cases with sequential FISH data, with the aim to emphasize the importance of continuous monitoring of the cytogenetic characteristics and shed light on the implications of cytogenetic clonal evolution in Chinese MM patients.

## Methods

### Patients and treatments

The patients who were diagnosed with NDMM at our hospital between January 2012 and December 2019 were retrospectively analyzed. One hundred patients who underwent at least twice fluorescence *in situ* hybridization (FISH) evaluations with intervals more than 3 months were included in the longitudinal subgroup. Meanwhile, 130 patients who received only once cytogenetic evaluation with complete clinical data were randomly selected with 15 percents of NDMM in the same period. The group consisted of 146 (63.5%) males and 84 (36.5%) females, with a median age of 61 years (30–83). The ISS stage I, II and III were counted 18.3, 38.2 and 43.5%, respectively. All patients were followed up for survival until March 31, 2022, with a median follow-up time of 41 (28–130) months from diagnosis. The baseline data at diagnosis was extracted from medical records, while follow-up information was recorded after each visit. This study was approved by the ethics committee of Peking University People’s Hospital.

The 230 patients received different regimens of initial therapy as follows, 162 (70.4%) patients were treated with bortezomib-based regimens, including BD (bortezomib, dexamethasone), BCD (bortezomib, cyclophosphamide, dexamethasone), and BAD (bortezomib, doxorubicin, dexamethasone). 41 (17.8%) patients received immunomodulator-based regimens, including RD (lenalidomide, dexamethasone), TAD (thalidomide, doxorubicin, dexamethasone) and TCD (thalidomide, cyclophosphamide, dexamethasone). 21 (9.1%) patients received bortezomib combined with immunomodulator regimens, including VTD (bortezomib, thalidomide and dexamethasone), VRD (bortezomib, lenalidomide and dexamethasone). 6 (2.6%) patients were treated with conventional VAD (vincristine, adriamycin, dexamethasone) chemotherapy. After induction therapy, 38 (16.5%) patients received first-line autologous stem cell transplantation (ASCT) as consolidation, and the others received lenalidomide, bortezomib or thalidomide plus dexamethasone as maintenance therapy.

#### ASCT

Patients underwent high-dose cyclophosphamide chemotherapy in combination with granulocyte colony-stimulating factor (G-CSF) for peripheral blood hematopoietic stem cell mobilization. The specific mobilization regimen was as follows: cyclophosphamide was administered intravenously over 2 days. Following chemotherapy, G-CSF was administered at a dose of 5–10 μg/(kg·d) to mobilize stem cells. Peripheral blood stem cell collection typically began on day 4–5 of G-CSF mobilization and continued for 1–2 days, with a maximum duration of 3 days. After collection, stem cells were reinfused electively, following pre-treatment with Mafran 2–3 days prior to reinfusion.

### Metaphase karyotype analysis and interphase FISH

A 24 h short-term culture and G-banding technique were routinely used for metaphase karyotyping in all 230 patients. At least 20 metaphase cells were analyzed as possible in each G-banding analysis and the karyotypes were described according to the International Nomenclature System of Human Cytogenetics (ISCN2020). All patients were analyzed for gain/amp *1q21, del(17p), del(13q)* and *IgH* rearrangement by iFISH on enrichment of CD138+ plasma cells which was performed by magnetic-activated cell sorting (MACS) (purchased by Miltenyi Biotec, Germany) using gene locus-specific probes (GLP) including GLP *1q21*, GLP *P53*, GLP *D13S391*, GLP *RB1*, GLP *IgH* at diagnosis. If an *IgH* rearrangement was suspected, dual-color and dual-fusion translocation probes such as *IGH/FGFR3*, *IGH/MAF* and *IGH/CCND1* were used for the detection of t(4;14)(p16;q32), t(14;16)(q32;q23) and t(11;14)(q13;q32) when the samples were available. Continuous FISH detections were performed in 100 patients during follow-up. In this study, for many patients with relatively stable disease following treatment, FISH assessments were typically conducted at regular intervals of 6 months to 1 year. However, for patients with disease progression, FISH re-evaluations were performed at any time. All probes were purchased from Peking GP Medical Technologies (Peking, China). At least 200 nuclei were counted for each probe with each sample, if the count value was near the threshold, the number of counted nuclei was increased to 500. The cut-off points for positive values (the mean of the normal control plus three standard deviations) were established in bone marrow from 20 healthy donors and 5.0% for gain/amp *1q21*, 8.0% for *D13S319* and *RB1* deletions, 8.0% for p53 deletion, 5.0% for *IgH* rearrangement and 3.0% for translocations.

### Definition and statistical analysis

The abnormalities of gain/amp(*1q21*), *del(17p)*, t(4;14), and t(14;16) identified by FISH were classified as HRA and the others were classified as non-HRA in this study. Among the patients with longitudinal FISH analysis, new emerging FISH abnormalities during follow-up were defined as “*de novo*” abnormalities. Cytogenetic clonal evolution was defined as any new acquired abnormality during follow-up. Treatment response was evaluated according to the international uniform response criteria (18). PFS was defined from the date of diagnosis to the date of death, disease progression, or the last follow-up. OS was defined from the date of diagnosis to the date of death or the last contact. The survival curves were generated using the Kaplan–Meier method and the survival comparisons were performed by the log-rank test. Fisher exact test were performed to make the comparison of categorical variables among groups. A *p*-value <0.05 was considered statistically significant. All *p*-values were two-sided. All statistical analyses were performed using SPSS version 22.0 (SPSS, Inc).

## Results

### Karyotyping and FISH results of 230 patients

Among the whole cohort of 230 patients, 219 (95.2%) had successful G-banding cytogenetic analysis at diagnosis including 159 (69.1%) with normal karyotypes and 60 (26.1%) with clonal abnormalities, 11 (4.8%) patients with failure karyotyping results or with less than 5 normal metaphases were not considered. Meanwhile, FISH was performed in all patients and revealed abnormalities in 180 (78.3%) patients, and the incidence of gain/amp *1q21*, *del(13q)*, *del(17p)*, and abnormal *IGH* were 47.8% (110/230), 42.6% (98/230), 6.1% (14/230), 63.5% (146/230), respectively. Among 146 patients with abnormal signal patterns by *IGH* break apart probes in whom *IGH* translocations were suspected, 135 (92.5%) were analyzed for t(11;14), t(4;14), and t(14;16), and the incidence of each translocation was 37.0% (50/135), 17.8% (24/135) and 2.2% (3/135). The cytogenetic characteristics in 230 patients at diagnosis were summarized in Table 1.

**Table 1:** Cytogenetic characteristics in 230 patients at diagnosis.

Cytogenetic characteristics	No. (%)
G-banding	*N* = 230
Normal karyotypes	159 (69.1%)
Unnormal karyotypes	60 (26.1%)
Complex karyotypes	36 (15.7%)
Less than 5 normal metaphases	11 (4.8%)
FISH	*N* = 230
Non-HRA	
*del(13q)*	108 (47%)
*IGH/CCND1*	55 (23.9%)
HRA	
*1q21*	111 (48.3%)
*del(17p)*	25 (10.9%)
*IGH/FGFR3*	29 (12.6%)
*IGH/MAF*	3 (1.3%)

FISH, fluorescence in situ hybridization; HRA, high-risk abnormalities.

### Cytogenetic clonal alterations

Continuous FISH detections were performed in 100 patients, and the results showed that 31 patients had unchanged FISH results during follow-up, including 10 with normal and 21 with abnormalities at diagnosis, and cytogenetic alterations were observed in 69 patients, out of whom 49 patients had *de novo* FISH abnormalities and 20 patients lost at least one or more previous existing abnormalities. Among 49 patients with *de novo* FISH abnormalities during follow-up, 26 patients had only 1 *de novo* abnormality while 23 patients had 2 or more new acquired abnormalities. According to the risk stratification, 35 patients acquired de novo HRA and 14 acquired non-HRA, and the new emerging aberrations included gain/amp (*1q21*) (25 cases), *del(13q)* (17 cases), *del(17p)* (11 cases), abnormal *IGH* (32 cases), *IGH::CCND1* (7 cases), *IGH::FGFR3* (6 cases) and *IGH::MAF* (1 case).

Among the 100 patients with continuous FISH detections, 67 patients underwent continuous G-banding analysis, and the results showed no change in 34 (50.8%) patients while 25 (37.3%) patients acquired new abnormalities and 8 (11.9%) lost at least one or more previous abnormalities.

Totally, regarding both G-banding and FISH results, 53% (53/100) of patients had cytogenetic evolution and the detailed clonal evolution types based on the initial and *de novo* FISH abnormalities and their prognostic risk stratification were listed in Table 2.

**Table 2:** Cytogenetic alterations in 100 patients with continuous FISH detections.

Cytogenetic alterations	No. (%)
G-banding	*N* = 67
Unchanged	34 (50.8%)
de novo abnormalities	25 (37.3%)
Loss at least one previous abnormalities	8 (11.9%)
FISH	*N* = 100
Unchanged	31 (31%)
de novo HRA	35 (35%)
de novo non-HRA	14 (14%)
Loss at least one previous abnormalities	20 (20%)
Clonal evolution types by FISH	*N* = 49
Initial HRA + de novo HRA	4 (8.2%)
Initial HRA + de novo non-HRA	5 (10.2%)
Initial non-HRA + de novo HRA	30 (61.2%)
Initial non-HRA + de novo non-HRA	10 (20.4%)

FISH, fluorescence in situ hybridization; HRA, high-risk abnormalities.

### Prognostic significance of the cytogenetic clonal evolution

#### Impact of cytogenetic clonal evolution on disease progression in MM patients: a longitudinal cytogenetic analysis

Among 100 patients with longitudinal FISH analysis, disease progression and death events were observed in 67 and 16 patients, respectively. It was observed that 83.7% (41/49) of patients with *de novo* FISH abnormalities suffered from disease progression, which was much higher than 56.9% (29/51) of those without *de novo* FISH aberrations (χ^2^ = 0.003). There was no significant difference on the frequencies of disease progression between the patients with *de novo* HRA and those with de novo non-HRA (84.8% vs. 62.5%, χ^2^ = 0.082), suggesting that de novo FISH abnormalities during follow-up were associated with disease progression regardless of new emerging HRA or non-HRA. Moreover, among 20 patients who experienced abnormalities loss after treatment during follow-up, 16 patients (80%) showed a good response to treatment (10 cases were evaluated as VGPR, and 6 cases were evaluated as CR), while 4 patients (20%) experienced disease progression. These findings suggested that the majority of patients with abnormalities loss demonstrated treatment efficacy.

As shown in Table 2, 100 MM patients underwent continuous cytogenetic analysis. Among them, 49 patients acquired new cytogenetic abnormalities, including 21 were treated with the BD/BCD regimen, and 28 received Non-BD/BCD regimens. Among the 51 patients without new acquired abnormalities, 28 were treated with the BD/BCD regimen, and 23 with Non-BD/BCD regimens. Statistical analysis revealed that regardless of the treatment regimens (whether BD/BCD or Non-BD/BCD), there was no significant difference in the probability of acquiring newly cytogenetic abnormalities (χ^2^ = 0.231), suggesting that the treatment regimen had no apparent effect on cytogenetic clonal evolution.

#### Impact of the number and timing of *de novo* FISH abnormalities during follow-up on survival outcomes

**Figure 1:**
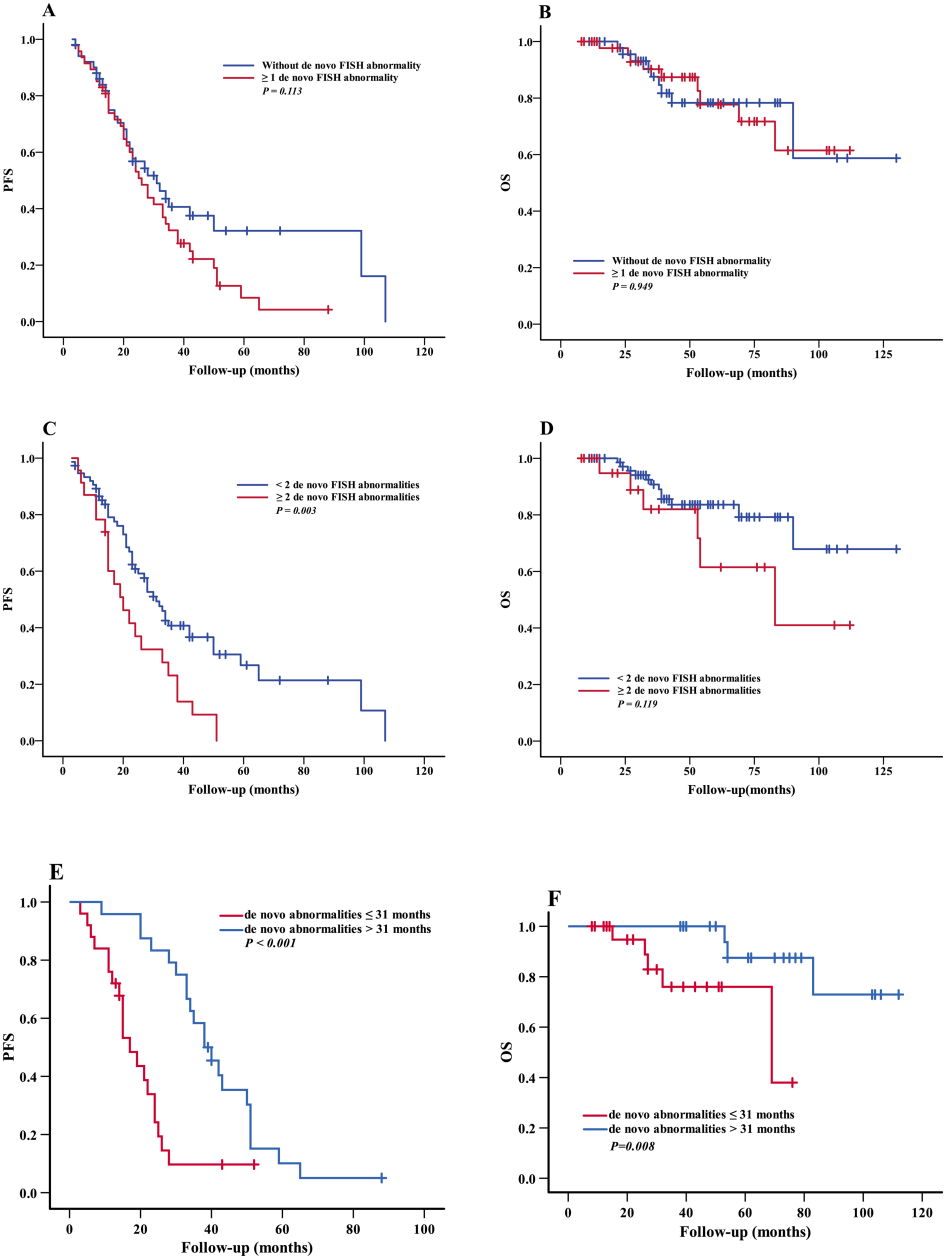
Impact of the number of de novo FISH abnormalities on survival. PFS **(A)** and OS **(B)** of patients with without de novo FISH abnormality and ≥ 1 de novo FISH abnormality. PFS **(C)** and OS **(D)** of patients with < 2 de novo FISH abnormalities and ≥ 2 de novo FISH abnormalities. PFS **(E)** and OS **(F)** of patients with de novo FISH abnormalities ≤ 31 months and > 31 months. OS, overall survival; PFS, progression-free survival.

Furthermore, we investigated the effect of the number of de novo FISH abnormalities on the survival, and the results showed that there were no significant difference in PFS (median 31 vs. 49 months, *p* = 0.113) (Figure 1A) and OS (median 90 vs. 101 months, *p* = 0.949) (Figure 1B) between the patients with ≥1 *de novo* FISH abnormality (49 cases) and those without *de novo* FISH abnormality (51 cases), whereas the patients with ≥2 de novo FISH abnormalities (23 cases) had an inferior PFS (median 24 vs. 45 months, *p* = 0.003) when compared to those with only 1 or no *de novo* FISH abnormality (77 cases) and there was no significant difference in OS between two groups (median 78 vs. 107 months, *p* = 0.119) (Figures 1C,D).

Among the 49 patients who developed de novo FISH abnormalities during follow-up, the median time to acquisition of new FISH abnormalities was 31 months (range: 4–71 months). We further analyzed the relationship between the timing of these abnormalities and survival outcomes. Our results indicated that patients who acquired new abnormalities within 31 months since diagnosis had significantly worse PFS (median: 20 vs. 41 months, *p* < 0.001) and OS (median: 61 vs. 100 months, *p* = 0.008) compared to those who acquired new abnormalities after 31 months (Figures 1E,F).

#### Impact of high risk abnormalities and treatment on survival

**Figure 2:**
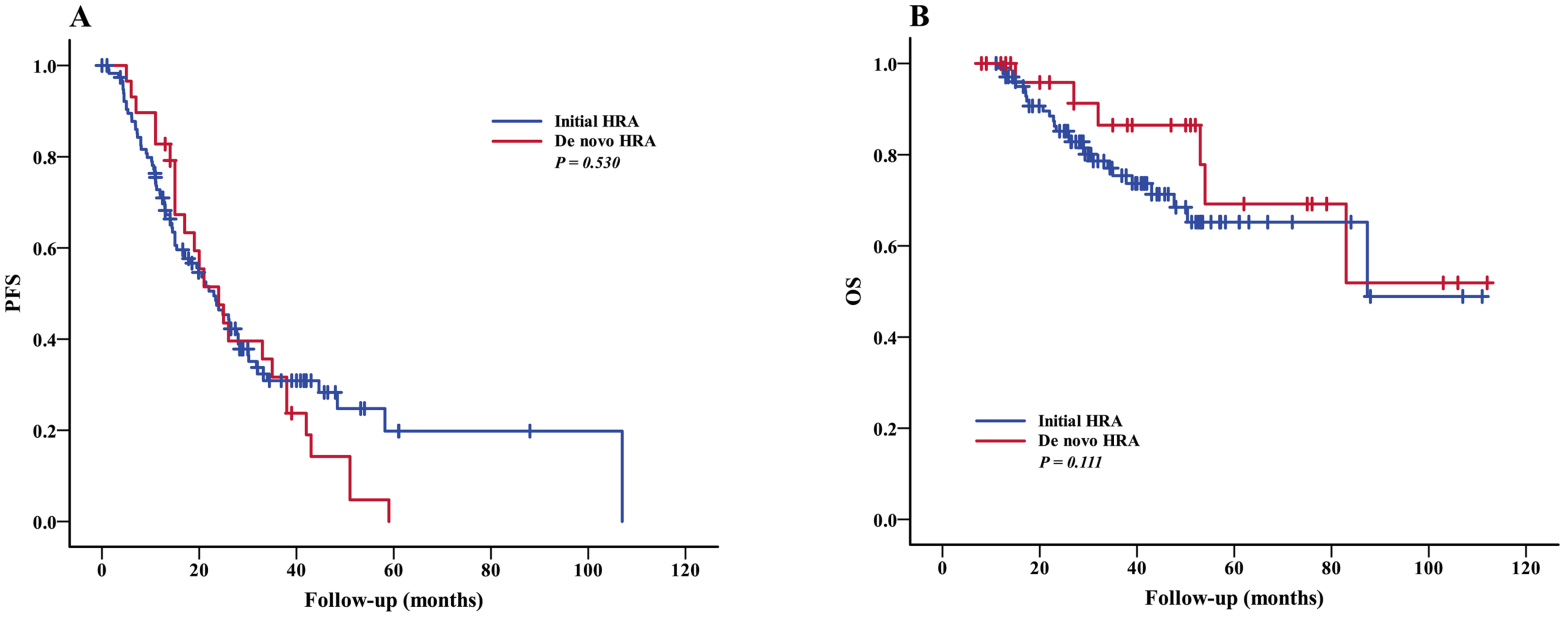
Impact of high risk abnormalities on survival. PFS **(A)** and OS **(B)** of patients with initial HRA and de novo HRA. OS, overall survival; PFS, progression-free survival; HRA, high-risk abnormalities.

To determine whether the initial HRA at diagnosis and *de novo* HRA during follow-up confer to different prognosis, patients with initial HRA and without de novo HRA during follow-up (120 cases) were defined as the initial HRA group, and patients with *de novo* HRA during follow-up and initial normal FISH (23 cases) or initial non-HRA (7 cases) were defined as the de novo HRA group. It was observed that there were no significant difference in PFS (median 38 vs. 27 months, *p* = 0.530) (Figure 2A) and OS (median 72 vs. 85 months, *p* = 0.111) (Figure 2B) between the initial HRA group and the de novo HRA group.

**Figure 3:**
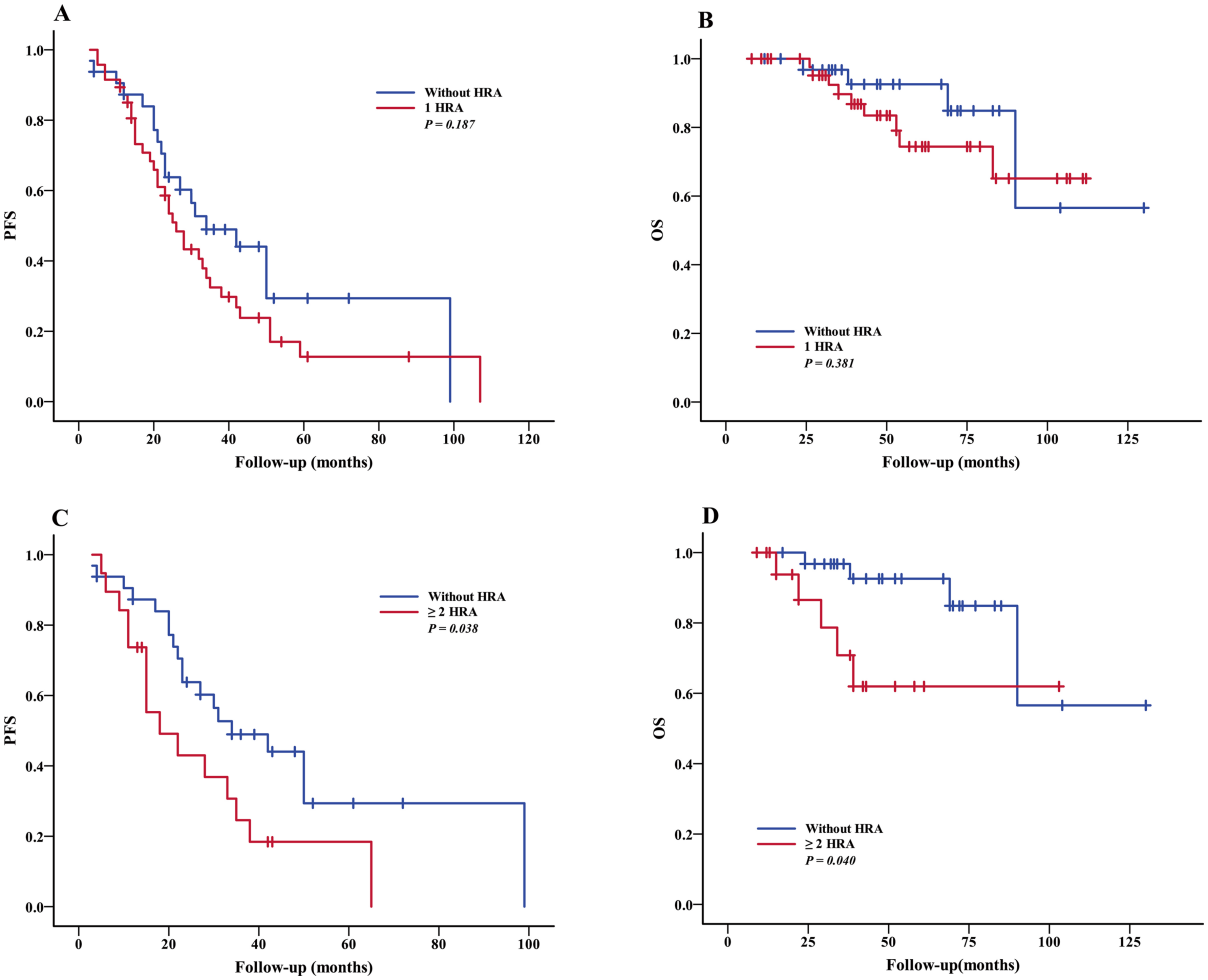
Impact of high risk abnormalities on survival. PFS **(A)** and OS **(B)** of patients without HRA and 1 HRA. PFS **(C)** and OS **(D)** of patients without HRA and 2 HRA. OS, overall survival; PFS, progression-free survival; HRA, high-risk abnormalities.

Among 100 patients with serial FISH analysis, considering the FISH results during the disease process, there were 48 cases with 1 HRA, 18 cases with 2 HRA, 1 case with 3 HRA, and 33 cases without HRA. Regarding the prognostic effect of the HRA number on the survival, the results showed that there were no significant difference in PFS (median 37 vs. 49 months, *p* = 0.187) (Figure 3A) and OS (median 91 vs. 107 months, *p* = 0.381) (Figure 3B) between the patients with 1 HRA and those without HRA. However, the patients with≥2 HRA (19 cases) had shorter PFS (median 28 vs. 49 months, *p* = 0.038) and OS (median 75 vs. 107 months, *p* = 0.040) when compared to those without HRA (33 cases) (Figures 3C,D).

Among the 100 MM patients who underwent continuous cytogenetic analysis, 11 patients received ASCT. Of the 49 patients who acquired new cytogenetic abnormalities, 5 underwent ASCT. Survival analysis indicated that there were no significant differences in PFS(median: 36 vs. 31 months, *p* = 0.705) and OS (median: 71 vs. 90 months, *p* = 0.471) between patients who underwent ASCT and those who received chemotherapy alone among the 49 patients.

## Discussion

The prognostic significance of baseline cytogenetic aberrations in NDMM is well-documented, which have been shown to have a significantly greater prognostic impact in MM than mutations in specific genes (19) and there is increasing evidence that the evolution of cytogenetic aberrations over time has an adverse effect on the prognosis of MM patients (20–22). The study from Aleksander et al. (23) showed that presence of clonal evolution, particularly the acquisition of new *del(17p)* at relapse negatively affect the outcome of MM, and similar results were observed in the Lakshman et al. (24) study. The study from Binder et al. (7) enrolled 989 MM patients including 304 with at least twice cytogenetic evaluations showed that the presence of t(11;14) at the time of diagnosis was associated with decreased odds of cytogenetic evolution during follow-up, while the presence of at least one trisomy or tetrasomy was associated with increased odds, and the development of additional abnormalities during the 3 years following diagnosis was associated with increased subsequent mortality. In addition, they also found that the prognostic significance of baseline cytogenetic abnormalities was most pronounced at the time of diagnosis and attenuated over time, the presence of cytogenetic high-risk features at diagnosis were associated with shorter OS but the presence of high-risk features were no longer associated with OS in those who survived 3 years after diagnosis, which highlighted the importance of continuous monitoring of cytogenetic characteristics and suggested that risk factors emerged at different times in the disease process may have different prognostic implications for MM patients. As more and more data suggest that disease progression, dissemination, and relapse in MM is driven by clonal evolution (25–28), an evolving consensus to reevaluate for cytogenetic high-risk features during follow-up has been reached, but the clinical implication of cytogenetic clonal evolution especially the prognostic significance of *de novo* HRA remains to be further clarified.

Our study revealed that 49% of Chinese MM patients acquired de novo FISH abnormalities during follow-up, and de novo FISH aberrations were associated with disease progression regardless of HRA or non-HRA (83.7% vs. 56.9%, *X^2^* = 0.003) and they were also conferred to an inferior median PFS (31 vs. 51 months, *p* = 0.032). In addition, the patients with 2 or more de novo FISH abnormalities had shorter median PFS (median 24 vs. 45 months, *p* = 0.003) when compared to those with one or no de novo FISH abnormality, and patients who acquired new abnormalities within 31 months since diagnosis had significantly worse PFS (median: 20 vs. 41 months, *p* < 0.001) and OS (median: 61 vs. 100 months, *p* = 0.008) compared to those who acquired new abnormalities after 31 months. Since clonal evolution may reflect the genomic instability, which is the hallmark of all neoplastic diseases and is the source of genetic heterogeneity of MM, it is reasonable to speculate that the more new emerging cytogenetic anomalies and the earlier new FISH abnormalities acquired, the greater the tumor instability and the worse the prognosis of MM. Consistent with this conjecture, our study showed that the higher number of *de novo* FISH abnormalities, the worse the survival, suggesting a cumulative adverse effect of the number of de novo FISH aberrations.

Although cytogenetic risk stratification of MM patients is widely used in clinical practice, there are some controversies about the prognostic impact of HRA in MM patients as new treatment strategies are constantly updated. Related study reported that with appropriately treatments, the survival of patients with certain high risk categories can approach that of patients with standard risk disease. In a large trial using bortezomib-based induction, early ASCT, and bortezomib maintenance, the median OS of patients with *del(17p)* was approximately 8 years (8-year survival rate of 52%), which was identical to patients with standard risk MM. In contrast, survival was lower for patients with t(4;14) (8-year survival rate, 33%) and for patients with gain(*1q21*) (8-year survival rate, 36%). These findings underscore the limitations of current risk stratification models in the context of modern therapy and highlight the need to stratify MM based on individual cytogenetic groups rather than arbitrary heterogeneous risk categories (29, 30). Considering the impact of the number of cytogenetic abnormalities on prognosis, Binder et al. (31) found that the greater the number of HRA at the time of diagnosis, the worse the prognosis of MM patients. In our study, almost all patients received modern therapies such as bortezomib, immunomodulator or ASCT as induction or maintenance, and no significant difference in PFS (median 37 vs. 49 months, *p* = 0.187) and OS (median 91 vs. 107 months, *p* = 0.381) were observed between the patients with 1 HRA and those without HRA, but the patients with ≥2 HRA had shorter median PFS (28 vs. 49 months, *p* = 0.038) and OS (75 vs. 107 months, *p* = 0.040) than the patients without HRA, suggesting by modern strategies of therapy, only two or more HRA were definitely adverse prognostic factor in Chinese MM patients, which highlighted the potential for risk stratification to change as treatments were updated.

In the era of new drugs, the role of ASCT has been questioned. However, ASCT remains the standard treatment recommended by international guidelines, including those of the American Society of Clinical Oncology and the European Society for Medical Oncology (32). Our study did not demonstrate that ASCT could improve the prognosis of high-risk patients (those who acquired new cytogenetic abnormalities during follow-up). We speculate that the limited number of patients undergoing ASCT in our cohort may have introduced statistical bias. In future studies, we plan to collect more cases to further explore this issue.

The ability to draw firm conclusions from our data is limited by the retrospective nature and a relatively small number of enrolled patients, but our results reaffirm the importance of continuous monitoring of the cytogenetic characteristics of MM during follow-up. *De novo* FISH abnormalities during follow-up are adverse prognostic factors in MM patients, especially when ≥2 new FISH anomalies are presented, and the presence of ≥2 HRA during the disease process are associated with poor survival in Chinese MM patients, which remains to be further confirmed in larger scale of studies.

## Conclusion

Re-evaluation of cytogenetic characteristics by serial FISH tests is important in MM patients. De novo FISH abnormalities during follow-up are adverse prognostic factors, especially when ≥2 new FISH anomalies and acquired new abnormalities within 31 months since diagnosis are presented, and the presence of ≥2 HRA during the disease process are associated with poor survival in Chinese MM patients.

## Data Availability

The original contributions presented in the study are included in the article/supplementary material, further inquiries can be directed to the corresponding author.
